# Comment on “Total Pancreatectomy With Islet Autotransplantation As an Alternative to High-Risk Pancreatojejunostomy After Pancreaticoduodenectomy: A Prospective Randomized Trial”

**DOI:** 10.1097/AS9.0000000000000246

**Published:** 2023-03-13

**Authors:** Leo Hans Bühler, Abraham Fingerhut

**Affiliations:** From the * Department of Surgery, University of Fribourg, Fribourg Cantonal Hospital, Fribourg, Switzerland; † Department of Surgery, Shanghai Jiao Tong University School of Medicine, Shanghai, China; ‡ Division for Surgical Research, Medical University of Graz, Graz, Austria.

We have read with interest the recent publication by Balzano et al,^1^ proposing total pancreatectomy with islet autotransplantation (TPIAT) as an alternative to high-risk pancreaticojejunostomy in patients undergoing pancreatic resection for neoplastic disease of the pancreatic head. The control group in the randomized trial included patients undergoing a classical pancreatoduodenectomy. Among the indications for resections listed in Supplemental Digital Content 1 (http://links.lww.com/SLA/E272) were patients with pancreatic ductal adenocarcinoma and various other neoplastic disorders.

Although the main endpoint in this study was the morbidity (number of patients with 1 or more complications), the authors also studied mortality in some detail and emphasized that that total pancreatectomy may prevent long-lasting decline of patients’ general condition associated with clinically relevant postoperative pancreatic fistula, and in particular, in patients with adenocarcinoma, where a complicated clinical course could impact oncological outcome (delay the start of adjuvant therapy). They even implied that (although as stated by the authors themselves, the study was underpowered to detect any statistically significant difference in survival), their therapeutic attitude may have contributed to the observed better survival in TPIAT (“a trend toward a reduction of mortality, even for patients with malignancy”).

We would kindly like to point out that (1) such a statement can be likened to “SPIN”^2^ and (2) in this study, the proportion of patients with ductal adenocarcinoma differed remarkedly between the groups: 29.0% (9/31) versus 16.7% (5/30) for the pancreaticoduodenectomy and total pancreatectomy groups, respectively. Even if the proper methodology were applied to this underpowered subgroup analysis,^3^ as overall survival would be highly dependent on the number of patients with pancreatic ductal adenocarcinoma, the outcome for the patients with pancreatic ductal adenocarcinoma versus patients with milder neoplastic diseases should be analyzed before implying that TPIAT could have a role in overall survival after resection for adenocarcinoma.

## Supplementary Material


