# Supplementary material for: Comment on “Total Pancreatectomy With Islet Autotransplantation As an Alternative to High-Risk Pancreatojejunostomy After Pancreaticoduodenectomy: A Prospective Randomized Trial”
Source: Ann Surg Open. 2023 Mar 13;4(1):e246. doi: 10.1097/AS9.0000000000000246 (PMC10431459; doi:10.1097/AS9.0000000000000246)
Supplement: Supplementary file 1 [file as9-4-e246-s001.docx]

**Supplementary Table 1: diseases of the pancreas in study patients**

|  | Group A  Pancreatoduodenectomy  with high-risk pancreatic anastomosis (n=31) | Group B  Total pancreatectomy  + IAT (n=30) | Group C  Pancreatoduodenectomy  with lower-risk pancreatic anastomosis (n=18) |
| --- | --- | --- | --- |
| Periampullary adenocarcinoma | 24 (77·4) | 21 (70) | 15 (83·3) |
| - Pancreatic ductal adenocarcinoma | 9 (29) | 5 (16·7) | 10 (55·6) |
| - Ampullary adenocarcinoma | 4 (12·9) | 3 (10) | 3 (16·7) |
| - Distal bile duct adenocarcinoma | 7 (22·6) | 9 (30) | 2 (11·1) |
| - Duodenum adenocarcinoma | 3 (9·7) | 4 (13·3) | 0 (0) |
| - Mixed Adenoneuroendocrine Carcinoma of the pancreas | 1 (3·2) | 0 (0) | 0 (0) |
| Other neoplastic, benign or borderline lesions | 7 (22·6) | 9 (30) | 3 (16·7) |
| - Leiomyosarcoma of the duodenum | 1 (3·2) | 0 (0) | 0 (0) |
| - Neuroendocrine neoplasm |  |  |  |
| - - G1 | 0 (0) | 0 (0) | 1 (5·6) |
| - - G≥2 | 2 (6·5) | 2 (6·7) | 0 (0) |
| - Duodenal gastrointestinal stromal tumor | 1 (3·2) | 0 (0) | 0 (0) |
| - Ampullary adenoma | 0 (0) | 2 (6·7) | 0 (0) |
| - Chronic pancreatitis | 1 (3·2) | 0 (0) | 0 (0) |
| - Distal bile duct pseudotumor | 0 (0) | 1 (3·3) | 0 (0) |
| - Gangliocytic paraganglioma | 0 (0) | 1 (3·3) | 0 (0) |
| - Mucinous cystadenoma | 2 (6·5) | 2 (6·7) | 2 (11·1) |
| - Serous cystoadenoma | 0 (0) | 1 (3·3) | 0 (0) |

**Supplementary Table 2. Islet isolation outcome and transplant characteristics stratified by study centres**

|  | TOT | Center 1 | Center 2 | p |
| --- | --- | --- | --- | --- |
| N | 30 (100) | 12 (100) | 18 (100) |  |
| Trimmed Pancreas Weight (g) | 75 (59-83) | 72 (63-84·7) | 76 (56·5-81·5) | 0·859 |
| Post purification IEQ | 170,000 (127,418-215,206) | 181,112 (137,146-226,475) | 147,175 (111,975-181,112) | 0·236 |
| Post-purification IEQ/g pancreas | 2,305 (1,847-3,148) | 2,381 (1,973-3,520) | 2,300 (1,806-2,899) | 0·268 |
| Islet transplanted | 29 (96·7) | 12 (100) | 17 (94·4) | 1 |
| Islet purification (%) | 45 (28·7-70) | 52·5 (22·5-67·5) | 42·5 (28·7-72·5) | 0·966 |
| Tissue volume (ml) | 1·5 (1-2·5) | 1·75 (1·05-2·59 | 1·5 (0·95-1·65) | 0·222 |
| Fresh/culture islet | 4/25 | 4/12 | 0/17 | 0·021 |
| Culture time (h) | 15 (13·4-16) | 16 (15-19·5) | 14 (12·7-15) | 0·014 |
| Recipient weight | 70 (65·5-75) | 70 (60·5-75) | 70 (66·5-88·5) | 0·305 |
| Recipient BMI | 25 (23·5-28·5) | 24 (22·25-26) | 28 (24-30) | 0·025 |
| Islet infused (IEQ/kg) | 1,863 (1,410-2,285) | 2,143 (1,668-2,722) | 1,627 (697-2,098) | 0·075 |
| Site (bone marrow/liver) | 2/27 | 2/10 | 0/17 | 0·163 |
| Δ portal vein pressure (cm H2O) | 0 (0-1) | 0 (0-1) | 0 (0-1) | 0·792 |
| Patients with complications related to islet infusion | 5 (17·2) | 2 (16·7) | 3 (17·6) | 1 |
| - Hepatic bleeding or portal thrombosis | 4 (13·8) | 2 (16·7) | 2 (11·8) | 1 |
| - Hemotorax | 1 (3·4) | 0 (0) | 1 (5·9) | 1 |

**Supplementary Table 3. Intraoperative and postoperative data**

|  |  |  |  |
| --- | --- | --- | --- |
|  | **Group A**  **Pancreatoduodenectomy**  **with high-risk pancreatic anastomosis** | **Group B**  **Total pancreatectomy**  **+ IAT** | **p** |
| N (%) | 31 (100) | 30 (100) |  |
| Intra-operative phase |  |  |  |
| Surgery duration (min) | 512 (391-604) | 443 (367-521) | 0·137 |
| Duct size (mm) | 2·4 (2-3) | 2·5 (2-3) | 0·492 |
| Blood loss (ml) | 500 (350-750) | 800 (450-950) | 0·086 |
| Blood transfusion [N(%)] | 4 (12·9) | 17 (56·7) | <0·001 |
| Blood volume (ml) | 1,125 (900-1570) | 900 (450-1350) | 0·229 |
| Plasma transfusion [N(%)] | 5 (16·1) | 5 (16·7) | 1 |
| Plasma volume (ml) | 520 (327-868) | 1,080 (585-1500) | 0·05 |
| Splenectomy [N(%)] | 0 (0) | 4 (13·3) | 0·053 |
| Pylorus preservation [N(%)] | 28 (90·3) | 26 (86·7) | 0·707 |
|  |  |  |  |
| Post-operative phase |  |  |  |
| Intensive care unit (ICU) [N(%)] | 7 (22·6) | 9 (30) | 0·57 |
| ICU duration (days) | 3 (1-8) | 1 (1-2·5) | 0·234 |
| Death within 90 days [N(%)] | 3 (9·7) | 1 (3·3) | 0·52 |
| Postoperative hospital stay (days) | 16 (12-30) | 10·5 (9-13·25) | <0·001 |
| Readmission after surgery [N(%)] | 11 (35·5) | 7 (23·3) | 0·402 |
| Time to readmission (days) | 22 (20-46) | 26 (12-29) | 0·338 |

**Supplementary Table 4. Comparison of complications after PD in patients with high-risk anastomosis (Group A) and patients intraoperatively excluded for lower-risk anastomosis (Group C)**

|  | Group A  Pancreatoduodenectomy  with high-risk pancreatic anastomosis  [N (%)] | Group C  Pancreatoduodenectomy  with lower-risk pancreatic anastomosis [N (%)] | p |
| --- | --- | --- | --- |
| N | 31 (100) | 18 (100) |  |
| Abdominal | 28 (90·3) | 12 (66·7) | 0·058 |
| Pancreatic fistula | 23 (74·2) | 11 (61·1) | 0·36 |
| - Grade A - Grade B - Grade C | 4 (17·4)  13 (56·5)  6 (26·1) | 6 (54·5)  3 (27·3)  2 (18·2) | 0·08 |
| Delayed gastric emptying | 6 (19·4) | 4 (22·2) | 1 |
| Post-pancreatectomy hemorrhage | 7 (22·6) | 1 (5·6) | 0·229 |
| Relaparotomy | 4 (12·9) | 2 (11·1) | 1 |
| Splenectomy | 0 (0) | 0 (0) | - |
| Duodeno-jejunal anastomosis leakage | 3 (9·7) | 0 (0) | 0·288 |
| Ileocolic anastomosis leakage | 2 (6·5) | 0 (0) | 0·526 |
| Biliary fistula | 5 (16·1) | 2 (11·1) | 1 |
| Lymphatic fistula | 0 (0) | 0 (0) | - |
| Abdominal fluid collections | 4 (12·9) | 3 (16·7) | 0·697 |
| Liver ischemia | 1 (3·2) | 0 (0) | 1 |
| Acute pancreatitis | 3 (9·7) | 0 (0) | 0·288 |
| Colangitis | 1 (3·2) | 0 (0) | 1 |
| Portal trombosis | 0 (0) | 0 (0) | - |
| Intestinal obstruction | 1 (3·2) | 0 (0) | 1 |
| Wound infection | 4 (12·9) | 0 (0) | 0·282 |
| Hepatic ematoma | 0 (0) | 0 (0) | - |
| Ascites | 1 (3·29) | 0 (0) | 1 |
| TOT abdominal complications | 65 | 23 |  |
|  |  |  |  |
| Other | 12 (38·7) | 6 (33·3) | 0·767 |
| Pneumonia | 2 (6·5) | 2 (11·1) | 0·618 |
| Sepsis | 6 (19·4) | 2 (11·1) | 0·693 |
| Arrhythmia | 1 (3·2) | 0 (0) | 1 |
| Embolism | 1 (3·2) | 0 (0) | 1 |
| Urinary retention | 0 (0) | 0 (0) | - |
| Urinary infection | 0 (0) | 2 (11·1) | 0·13 |
| Pleural effusion | 2 (6·5) | 0 (0) | 0·526 |
| Pancytopenia | 1 (3·2) | 0 (0) | 1 |
| Acute peripheral arterial occlusion | 1 (3·2) | 0 (0) | 1 |
| Jugular vein thrombosis | 1 (3·2) | 0 (0) | 1 |
| Stroke | 1 (3·2) | 0 (0) | 1 |
| TOT other complications | 16 | 6 |  |
|  |  |  |  |
| TOT complications | 81 | 29 |  |

**Supplementary Table 5. Group A vs Group C univariate logistic regression analysis of complications adjusted for age and sex**

|  | ITT |  |  |  |  |
| --- | --- | --- | --- | --- | --- |
|  | **Odd ratio** | **95% C.I.** | | **p** |  |
| Any complications | 3·162 | 0·576 | 17·34 | 0·185 |  |
| Abdominal complications | 4·99 | 1·01 | 24·4 | 0·048 |  |
| Other complications | 1·429 | 0·405 | 5·043 | 0·579 |  |
| At least 2 complications | 3·048 | 0·86 | 10·8 | 0·084 |  |
| Complications with grade ≥2 | 12·298 | 1·9 | 79 | 0·008 |  |
| Complications with grade ≥3 | 3·275 | 0·829 | 12·93 | 0·09 |  |
| Grade B-C fistula | 4·553 | 1·229 | 16·860 | 0·023 |  |
| Relaparotomy | 1·406 | 0·219 | 9·031 | 0·72 |  |
| Readmission after surgery | 2·744 | 0·621 | 12·136 | 0·183 |  |

**
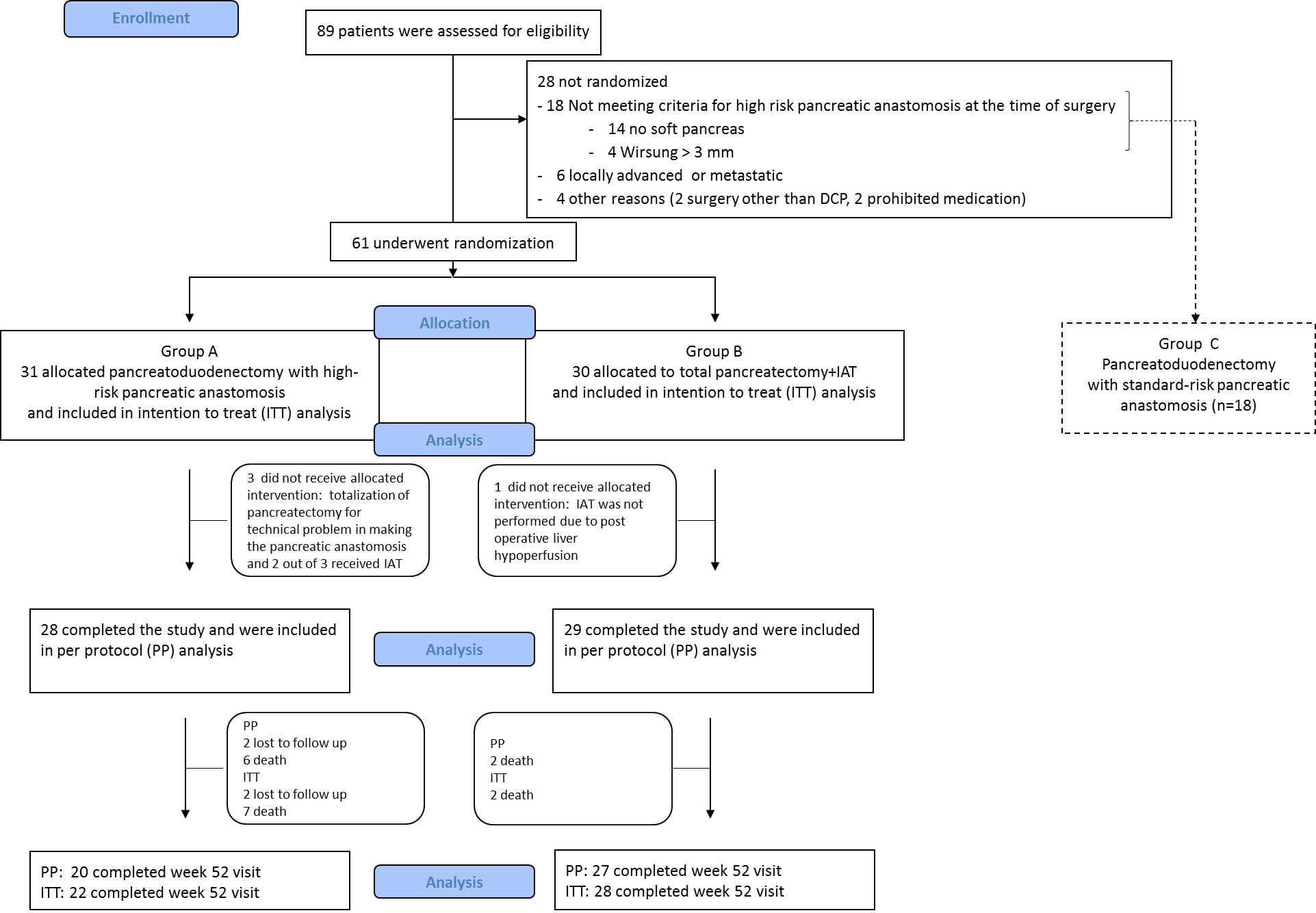
**

**Supplementary Figure 1. Patient disposition flow chart showing patient recruitment, random assignment to treatment, and discontinuations.**

**
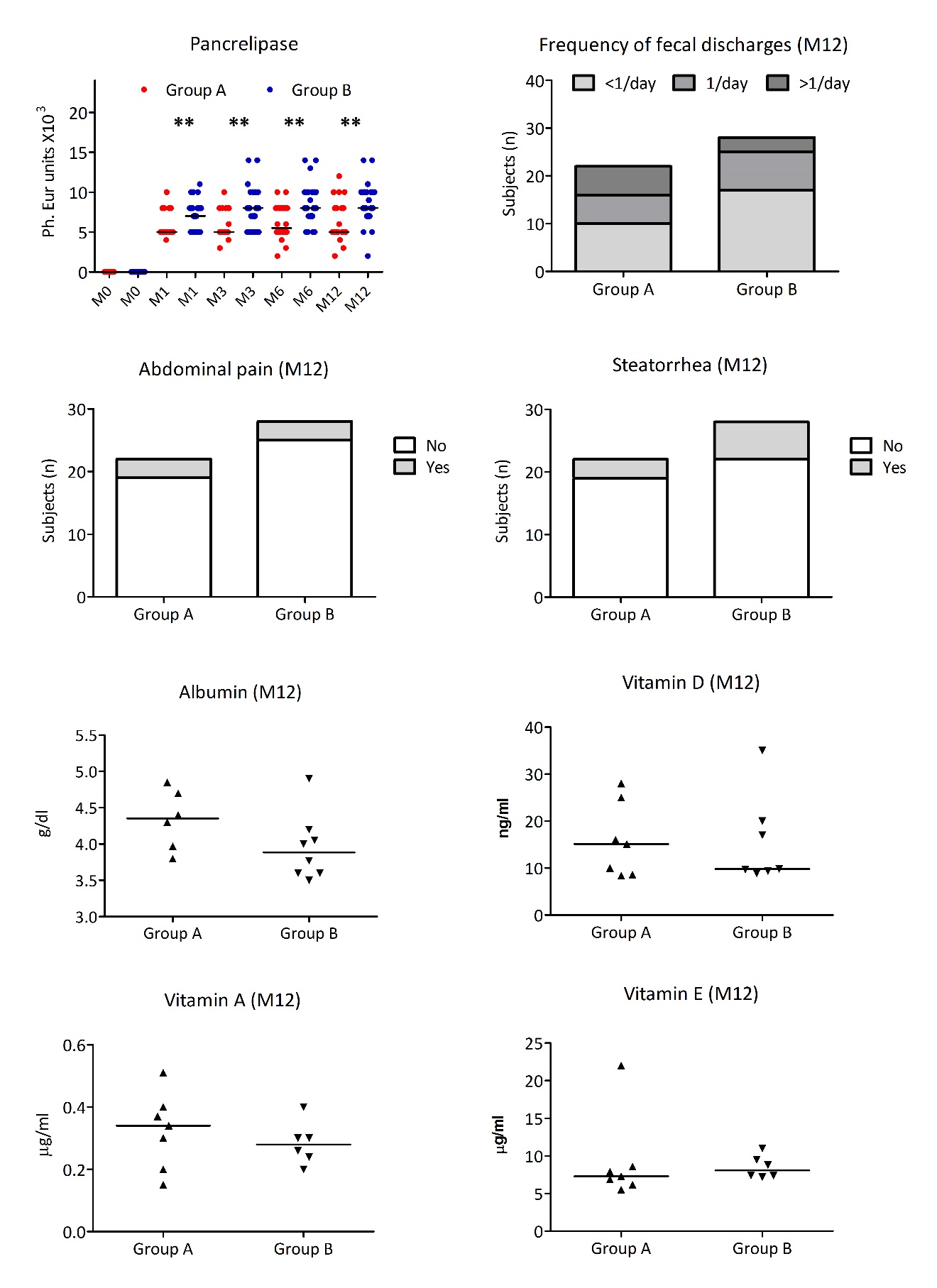
Supplementary Figure 2. Exocrine function after pancreatic surgery.** Pancreatic enzyme replacement therapy is reported as dot plot before and at month 1, 3, 6 and 12 after surgery. Symptoms of exocrine insufficiency, serum levels of albumin and fat-soluble vitamins are reported as histogram and dot plot, respectively, at month 12 (M12) after surgery. Group A consists of 20 patients assigned to pancreaticoduodenectomy with pancreatic anastomosis who completed week 52 visit. Group B consists of 17 patients assigned to total pancreatectomy with islet autotransplantation who completed week 52 visit. Analysis were performed by two-sided Fisher’s exact test or Mann Whitney test. *<0·05, **<0·01.

**
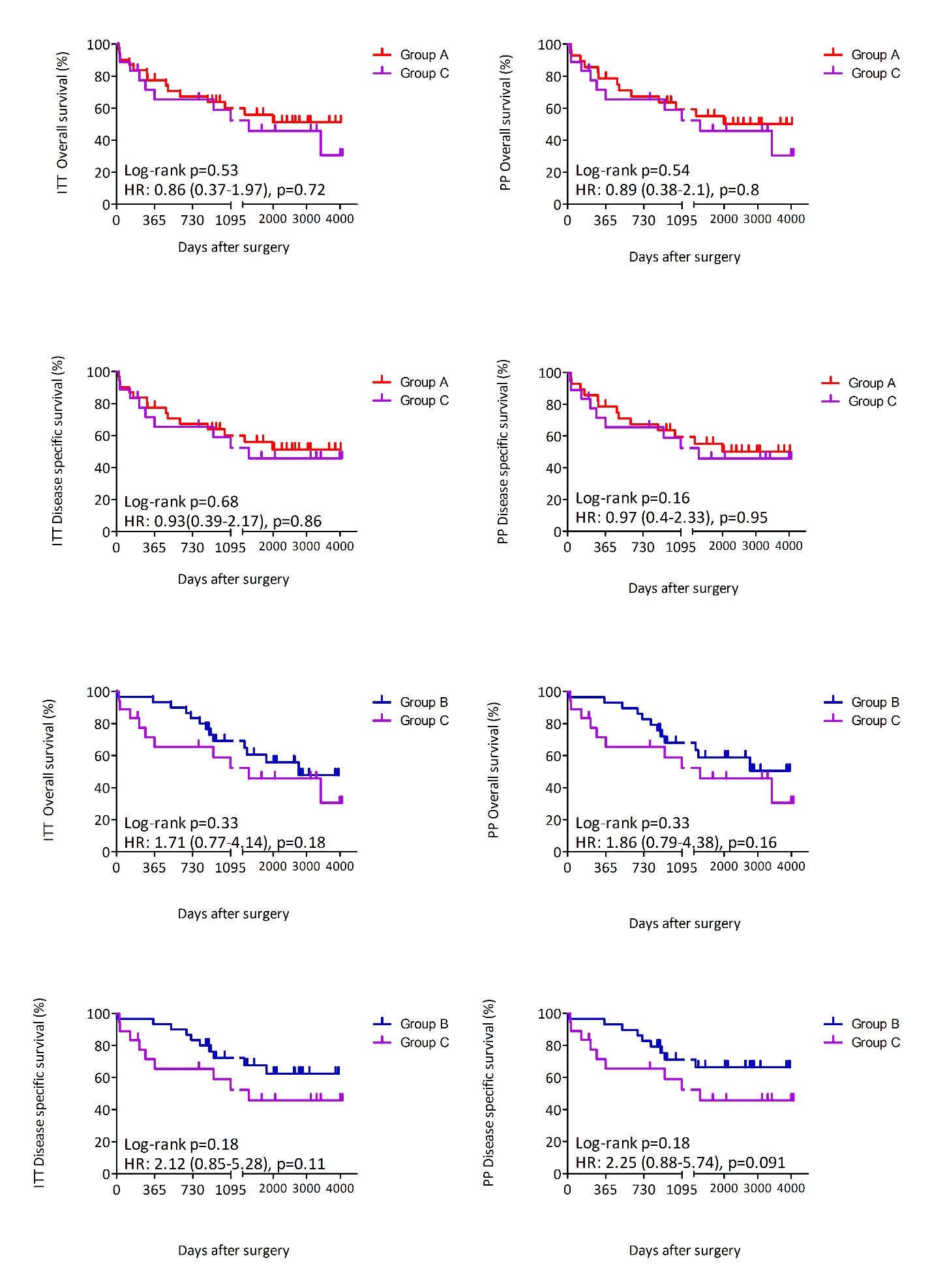
**

**Supplementary Figure 3. Patient survival.** Intention to treat (ITT) and per protocol (PP) probability of overall and disease specific survival after surgery, according to Kaplan-Meier. Analysis was performed by Log-rank (Mantel-Cox) test. The Univariate hazard ratios (HRs) adjusted for age and sex were reported.

**
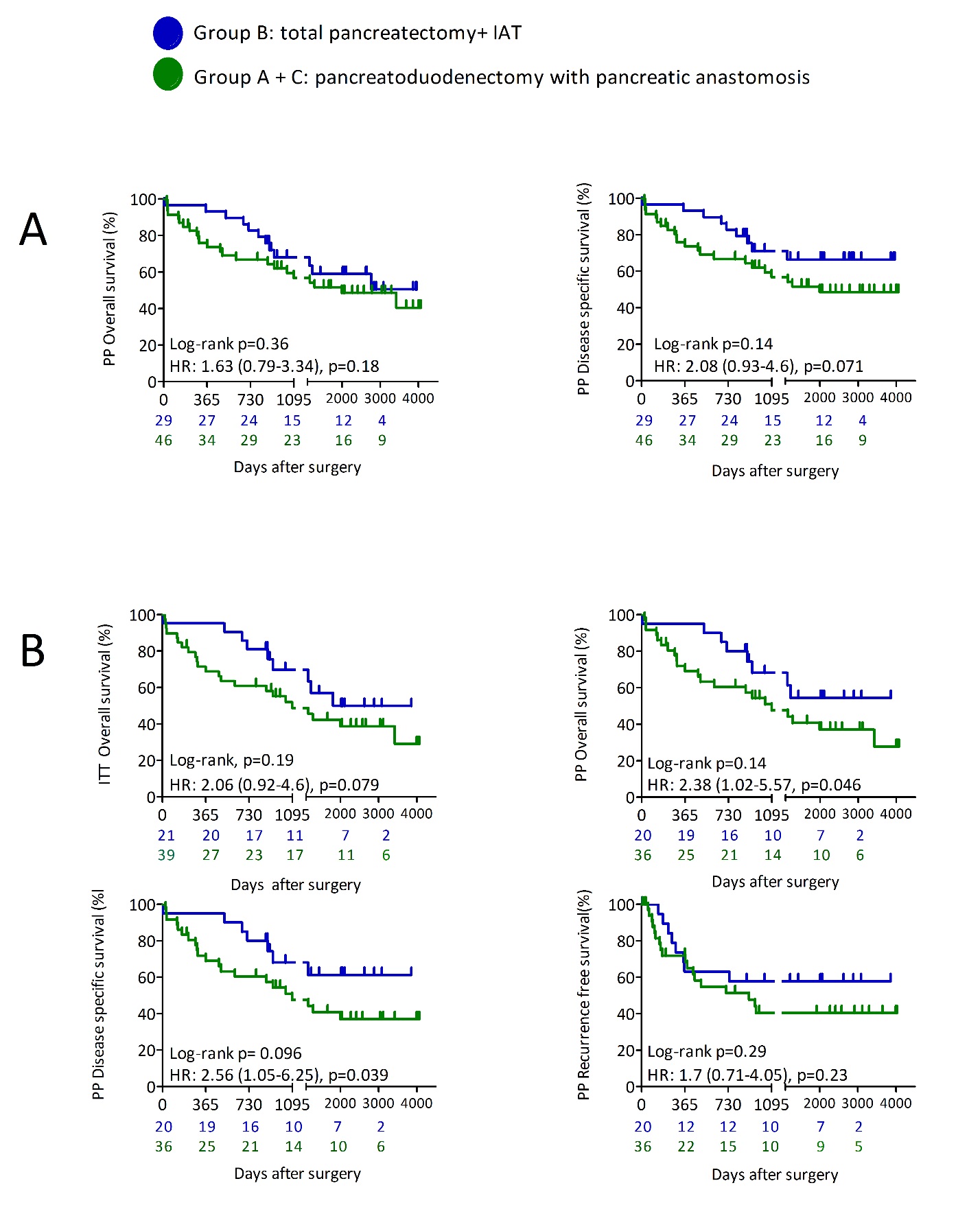
**

**Supplementary Figure 4. Patient survival and oncologic follow-up.** *Panel A*. Per protocol (PP) probability of overall and disease specific survival after surgery, according to Kaplan-Meier. *Panel B*. Intention to treat (ITT) and per protocol (PP) probability of overall, PP disease-specific, PP disease-free free survival after surgery in subjects with epithelial malignancy, according to Kaplan-Meier. Analysis was performed by Log-rank (Mantel-Cox) test. The Univariate hazard ratios (HRs) adjusted for age and sex (and tumor grade in patients with malignant neoplasm) were reported.
